# Tris(tetra­methyl­ammonium) tetra-μ_2_-sulfido-tetra­sulfidocopper(I)dimolyb­denum(VI) *N*,*N*-dimethyl­formamide solvate

**DOI:** 10.1107/S1600536808036532

**Published:** 2008-11-13

**Authors:** Yuan Cao, Yulin Chen, Guosheng Cheng

**Affiliations:** a Institute of Transition Metallic Compounds, Nanjing University of Information Science and Technology, 219 Ninliu Road, Nanjing 210044, Jiangsu, People’s Republic of China; b School of Mathematics and Physics, Nanjing University of Information Science and Technology, 219 Ninliu Road, Nanjing 210044, Jiangsu, People’s Republic of China

## Abstract

The title compound, (C_4_H_12_N)_3_[CuMo_2_S_8_]·C_3_H_7_NO, was obtained from the self-assembly of tetra­thio­molybdate, tetra­methyl­ammonium nitrate and cuprous sulfide in dimethyl­formamide (DMF). The asymmetric unit contains three (NMe_4_)^+^ cations, one [Mo_2_S_8_Cu]^3−^ anion and one DMF solvent mol­ecule, and no obvious inter­actions are observed between these species. The trinuclear anion can be viewed as fused [MoS_4_Cu]^−^ units sharing a copper center. The geometric parameters of the trivalent anion are comparable to those reported for other related salts including isomorphous anions, namely (NEt_4_)_2_(PPh_4_)[Mo_2_S_8_Cu] (*a*) and (Ph_3_P=N=PPh_3_)_2_(NEt_4_)[W_2_S_8_Cu]·2CH_3_CN (*b*). However, the Mo—Cu—Mo angle is found to be 160.24 (3)° for the title salt, while this angle is 162.97 (2)° in (*a*) and the W—Cu—W angle is 170.3 (2)° in (*b*), indicating that the largest deviation from linearity is in the title compound.

## Related literature

For related Mo^VI^/Cu^I^ and W^VI^/Cu^I^ complexes, see: Niu *et al.* (2002 ▶); Maiti *et al.* (2004 ▶); Müller *et al.* (1989 ▶).
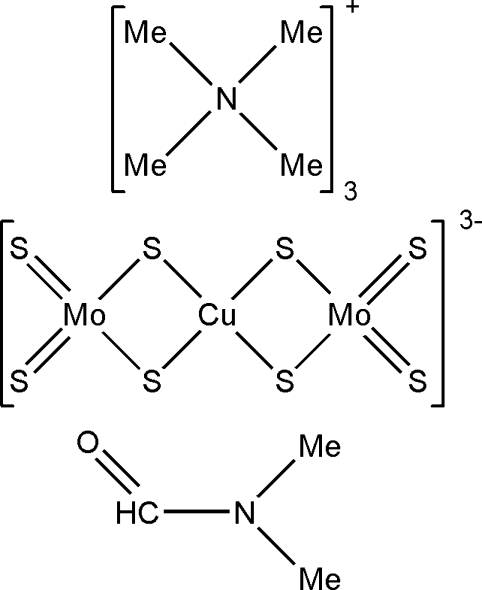


## Experimental

### 

#### Crystal data

(C_4_H_12_N)_3_[CuMo_2_S_8_]·C_3_H_7_NO
*M* *_r_* = 807.43
Monoclinic, 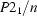

*a* = 9.4380 (19) Å
*b* = 20.336 (4) Å
*c* = 17.718 (4) Å
β = 98.60 (3)°
*V* = 3362.4 (12) Å^3^
*Z* = 4
Mo *K*α radiation
μ = 1.87 mm^−1^
*T* = 293 (2) K
0.40 × 0.30 × 0.25 mm

#### Data collection

Bruker APEX CCD area-detector diffractometer
Absorption correction: multi-scan (*SADABS*; Bruker, 1998 ▶) *T*_min_ = 0.527, *T*_max_ = 0.618
17155 measured reflections
6625 independent reflections
5061 reflections with *I* > 2σ(*I*)
*R*_int_ = 0.039

#### Refinement

*R*[*F* ^2^ > 2σ(*F* ^2^)] = 0.042
*wR*(*F* ^2^) = 0.136
*S* = 1.05
6625 reflections
274 parameters
H-atom parameters constrained
Δρ_max_ = 0.93 e Å^−3^
Δρ_min_ = −0.85 e Å^−3^

### 

Data collection: *SMART* (Bruker, 1998 ▶); cell refinement: *SAINT* (Bruker, 1998 ▶); data reduction: *SAINT*; program(s) used to solve structure: *SHELXTL* (Sheldrick, 2008 ▶); program(s) used to refine structure: *SHELXTL*; molecular graphics: *SHELXTL*; software used to prepare material for publication: *SHELXTL*.

## Supplementary Material

Crystal structure: contains datablocks I, global. DOI: 10.1107/S1600536808036532/bh2199sup1.cif

Structure factors: contains datablocks I. DOI: 10.1107/S1600536808036532/bh2199Isup2.hkl

Additional supplementary materials:  crystallographic information; 3D view; checkCIF report

## Figures and Tables

**Fig. 1.**
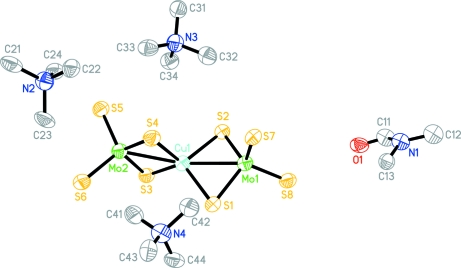
The molecular structure of the title compound, with atom labels and 50% probability displacement ellipsoids. All H atoms have been omitted.

## supplementary crystallographic information

### Comment

The title compound, (NMe_4_)_3_[Mo_2_S_8_Cu].C_3_H_7_ON, was obtained from the self-assembly of tetrathiomolybdate, tetramethylammonium nitrate and cuprous sulfide in *N*,*N*-dimethylformamide (DMF). The asymmetric unit contains three tetramethylammonium cations, one [Mo_2_S_8_Cu]^3-^ anion and one DMF solvent molecule, with no obvious interactions observed among these species. The trinuclear anion can be viewed as fused by two binuclear units [MoS_4_Cu]^-^ through one shared copper. The two binuclear units are perpendicular to each other, as found in other linear trinuclear clusters, such as {MoS_4_Cu_2_[PPh_2_(py)]_4_} (py = pyridyl) (Niu *et al.*, 2002). However, in the latter, [MoS_4_]^2-^ is a tetradentate ligand, while in the former, it serves as a bidentate ligand. In the title cluster, one Mo center is close to retain the original tetrahedral configuration of free [MoS_4_]^2-^, with six S—Mo—S bond angles varying from 108.88 (6) to 111.20 (6)°. The other Mo center distorts slightly more, the corresponding angles ranging from 107.24 (6) to 111.79 (6)°. The geometric parameters of the trivalent anion are comparable to those reported for the compounds bis(tetraethylammonium) tetraphenylphosphonium bis(di-µ_2_-sulfido-dithioxomolybdenum)copper (*a*) (Maiti *et al.*, 2004) and bis[bis(triphenylphosphine)iminium] tetraethylammonium bis(di-µ_2_-sulfido-dithioxotungsten)copper acetonitrile disolvate (*b*) (Müller *et al.*, 1989), except a notable difference among the Mo—Cu—Mo angles: 160.24 (3)° for the title compound, 162.97 (2)° for (*a*), while W—Cu—W angle is 170.3 (2)° in (*b*), indicating a largest deviation from ideal linear array in the title salt.

### Experimental

0.75 mmol of Cu_2_S powder was added to a solution of [NH_4_]_2_MoS_4_ (1 mmol in 10 ml DMF) and stirred for 4 h. at room temperature. After filtration, the filtrate was carefully laid on the surface with DMF (3 ml) and saturated (Me_4_N)NO_3_ solution (5 ml in methanol), successively. Black red polyhedric crystals of the title compound were obtained after two weeks.

### Refinement

All H atoms were positioned geometrically and refined using a riding model, with C—H = 0.93 Å for aldehydic H atom and C—H = 0.96 Å for methyl groups. Isotropic displacement parameters for H atoms were calculated as *U*_iso_(H) = 1.5*U*_eq_(carrier C), except for H11A: *U*_iso_(H11A) = 1.2*U*_eq_(C11).

### Crystal data

**Table:**

(C_4_H_12_N)_3_[CuMo_2_S_8_]·C_3_H_7_NO	*F*_000_ = 1640
*M**_r_* = 807.43	*D*_x_ = 1.595 Mg m^−^^3^
Monoclinic, *P*2_1_/*n*	Mo *K*α radiation λ = 0.71073 Å
Hall symbol: -P 2yn	Cell parameters from 7032 reflections
*a* = 9.4380 (19) Å	θ = 1.2–26.1º
*b* = 20.336 (4) Å	µ = 1.87 mm^−^^1^
*c* = 17.718 (4) Å	*T* = 293 (2) K
β = 98.60 (3)º	Polyhedron, black red
*V* = 3362.4 (12) Å^3^	0.40 × 0.30 × 0.25 mm
*Z* = 4	

### Data collection

**Table:**

Bruker APEX CCD area-detector diffractometer	6625 independent reflections
Radiation source: fine-focus sealed tube	5061 reflections with *I* > 2σ(*I*)
Monochromator: graphite	*R*_int_ = 0.039
*T* = 293(2) K	θ_max_ = 26.0º
φ and ω scans	θ_min_ = 1.5º
Absorption correction: multi-scan(SADABS; Bruker, 1998)	*h* = −11→11
*T*_min_ = 0.527, *T*_max_ = 0.618	*k* = −24→25
17155 measured reflections	*l* = −21→21

### Refinement

**Table:**

Refinement on *F*^2^	Secondary atom site location: difference Fourier map
Least-squares matrix: full	Hydrogen site location: inferred from neighbouring sites
*R*[*F*^2^ > 2σ(*F*^2^)] = 0.042	H-atom parameters constrained
*wR*(*F*^2^) = 0.136	*w* = 1/[σ^2^(*F*_o_^2^) + (0.0646*P*)^2^] where *P* = (*F*_o_^2^ + 2*F*_c_^2^)/3
*S* = 1.05	(Δ/σ)_max_ < 0.001
6625 reflections	Δρ_max_ = 0.93 e Å^−^^3^
274 parameters	Δρ_min_ = −0.85 e Å^−^^3^
Primary atom site location: structure-invariant direct methods	Extinction correction: none

### Fractional atomic coordinates and isotropic or equivalent isotropic displacement parameters (Å^2^)

**Table:**

	*x*	*y*	*z*	*U*_iso_*/*U*_eq_	
Mo1	0.25496 (5)	0.15816 (2)	0.93899 (3)	0.04775 (14)	
Mo2	0.31519 (5)	0.05342 (2)	0.66754 (3)	0.04773 (14)	
Cu1	0.24065 (7)	0.09541 (3)	0.80223 (4)	0.04912 (18)	
S1	0.11495 (15)	0.18682 (7)	0.83342 (8)	0.0494 (3)	
S2	0.31587 (15)	0.05454 (7)	0.92589 (8)	0.0489 (3)	
S3	0.12487 (15)	0.02298 (7)	0.71839 (8)	0.0483 (3)	
S4	0.43472 (16)	0.12836 (7)	0.74081 (8)	0.0514 (3)	
S5	0.45325 (16)	−0.03117 (8)	0.65994 (8)	0.0532 (3)	
S6	0.24512 (16)	0.09507 (7)	0.55703 (9)	0.0543 (3)	
S7	0.44848 (16)	0.21739 (7)	0.95638 (8)	0.0516 (3)	
S8	0.13280 (16)	0.16860 (7)	1.03204 (8)	0.0522 (3)	
O1	0.1656 (5)	0.1069 (2)	1.2555 (3)	0.0610 (11)	
N1	0.2487 (5)	0.1922 (2)	1.3279 (3)	0.0500 (11)	
C11	0.2068 (7)	0.1295 (3)	1.3196 (4)	0.0553 (14)	
H11A	0.2088	0.1028	1.3624	0.066*	
C12	0.2912 (7)	0.2217 (3)	1.4013 (4)	0.0605 (15)	
H12A	0.3093	0.1879	1.4393	0.091*	
H12B	0.2160	0.2499	1.4133	0.091*	
H12C	0.3767	0.2471	1.4006	0.091*	
C13	0.2171 (7)	0.2367 (3)	1.2639 (4)	0.0579 (15)	
H13A	0.1901	0.2118	1.2179	0.087*	
H13B	0.3006	0.2625	1.2592	0.087*	
H13C	0.1398	0.2653	1.2719	0.087*	
N2	0.7731 (5)	0.1115 (2)	0.5453 (3)	0.0507 (11)	
C21	0.8726 (7)	0.1130 (3)	0.4883 (4)	0.0616 (16)	
H21A	0.9607	0.0916	0.5089	0.092*	
H21B	0.8918	0.1578	0.4761	0.092*	
H21C	0.8301	0.0905	0.4429	0.092*	
C22	0.8482 (7)	0.1530 (3)	0.6068 (3)	0.0549 (14)	
H22A	0.9399	0.1340	0.6254	0.082*	
H22B	0.7922	0.1557	0.6476	0.082*	
H22C	0.8613	0.1963	0.5873	0.082*	
C23	0.6306 (7)	0.1439 (3)	0.5187 (4)	0.0600 (15)	
H23A	0.5717	0.1411	0.5583	0.090*	
H23B	0.5839	0.1219	0.4738	0.090*	
H23C	0.6457	0.1892	0.5070	0.090*	
C24	0.7503 (7)	0.0474 (3)	0.5762 (4)	0.0557 (14)	
H24A	0.8411	0.0269	0.5931	0.084*	
H24B	0.6960	0.0207	0.5376	0.084*	
H24C	0.6985	0.0519	0.6186	0.084*	
N3	0.7749 (5)	0.0309 (2)	0.8680 (3)	0.0525 (11)	
C31	0.9019 (7)	−0.0048 (3)	0.9017 (3)	0.0554 (14)	
H31A	0.9818	0.0248	0.9108	0.083*	
H31B	0.9224	−0.0389	0.8674	0.083*	
H31C	0.8857	−0.0241	0.9491	0.083*	
C32	0.7374 (7)	0.0836 (3)	0.9250 (4)	0.0574 (14)	
H32A	0.8146	0.1146	0.9350	0.086*	
H32B	0.7231	0.0626	0.9719	0.086*	
H32C	0.6513	0.1061	0.9036	0.086*	
C33	0.7972 (6)	0.0578 (3)	0.7979 (4)	0.0577 (14)	
H33A	0.8760	0.0881	0.8061	0.087*	
H33B	0.7123	0.0805	0.7751	0.087*	
H33C	0.8187	0.0232	0.7645	0.087*	
C34	0.6474 (4)	−0.01784 (16)	0.85381 (19)	0.0594 (14)	
H34A	0.6675	−0.0512	0.8185	0.089*	
H34B	0.5620	0.0054	0.8329	0.089*	
H34C	0.6339	−0.0380	0.9012	0.089*	
N4	0.2179 (4)	0.32703 (16)	0.67898 (19)	0.0504 (10)	
C41	0.2608 (4)	0.27407 (16)	0.63794 (19)	0.0593 (15)	
H41A	0.2482	0.2339	0.6647	0.089*	
H41B	0.3599	0.2791	0.6325	0.089*	
H41C	0.2037	0.2727	0.5883	0.089*	
C42	0.3149 (6)	0.3291 (3)	0.7528 (3)	0.0522 (13)	
H42A	0.3032	0.2897	0.7811	0.078*	
H42B	0.2922	0.3666	0.7816	0.078*	
H42C	0.4124	0.3322	0.7436	0.078*	
C43	0.2388 (6)	0.3897 (3)	0.6357 (3)	0.0512 (13)	
H43A	0.3368	0.3929	0.6276	0.077*	
H43B	0.2152	0.4269	0.6647	0.077*	
H43C	0.1774	0.3890	0.5873	0.077*	
C44	0.0667 (6)	0.3221 (3)	0.6915 (4)	0.0560 (14)	
H44A	0.0536	0.2824	0.7190	0.084*	
H44B	0.0053	0.3214	0.6432	0.084*	
H44C	0.0431	0.3593	0.7206	0.084*	

### Atomic displacement parameters (Å^2^)

**Table:**

	*U*^11^	*U*^22^	*U*^33^	*U*^12^	*U*^13^	*U*^23^
Mo1	0.0462 (3)	0.0487 (3)	0.0477 (3)	0.00610 (19)	0.00487 (19)	−0.00193 (19)
Mo2	0.0459 (3)	0.0490 (3)	0.0484 (3)	0.00548 (19)	0.00763 (19)	0.00304 (19)
Cu1	0.0491 (4)	0.0505 (4)	0.0476 (4)	0.0071 (3)	0.0065 (3)	−0.0035 (3)
S1	0.0479 (7)	0.0533 (7)	0.0479 (7)	0.0088 (6)	0.0104 (6)	−0.0037 (6)
S2	0.0476 (7)	0.0524 (7)	0.0464 (7)	0.0074 (6)	0.0060 (5)	−0.0030 (6)
S3	0.0467 (7)	0.0504 (7)	0.0484 (7)	0.0073 (5)	0.0092 (6)	0.0045 (6)
S4	0.0545 (8)	0.0472 (7)	0.0531 (7)	0.0107 (6)	0.0103 (6)	0.0061 (6)
S5	0.0503 (8)	0.0580 (8)	0.0526 (7)	0.0073 (6)	0.0124 (6)	0.0045 (6)
S6	0.0538 (8)	0.0518 (8)	0.0567 (8)	0.0112 (6)	0.0069 (6)	−0.0029 (6)
S7	0.0513 (7)	0.0521 (7)	0.0526 (7)	0.0119 (6)	0.0118 (6)	−0.0056 (6)
S8	0.0511 (7)	0.0520 (7)	0.0540 (8)	0.0122 (6)	0.0101 (6)	0.0052 (6)
O1	0.058 (2)	0.069 (3)	0.056 (2)	0.014 (2)	0.0089 (19)	0.022 (2)
N1	0.046 (2)	0.050 (3)	0.055 (3)	−0.0102 (19)	0.011 (2)	0.012 (2)
C11	0.053 (3)	0.053 (3)	0.061 (4)	−0.015 (3)	0.010 (3)	0.015 (3)
C12	0.062 (4)	0.053 (3)	0.068 (4)	−0.014 (3)	0.014 (3)	0.010 (3)
C13	0.063 (3)	0.044 (3)	0.061 (3)	0.009 (3)	−0.006 (3)	−0.015 (3)
N2	0.051 (3)	0.051 (3)	0.051 (3)	−0.005 (2)	0.011 (2)	0.011 (2)
C21	0.060 (4)	0.056 (3)	0.061 (3)	0.017 (3)	−0.015 (3)	0.008 (3)
C22	0.051 (3)	0.067 (4)	0.050 (3)	0.005 (3)	0.017 (2)	0.010 (3)
C23	0.067 (4)	0.051 (3)	0.054 (3)	0.020 (3)	−0.015 (3)	0.008 (3)
C24	0.054 (3)	0.059 (3)	0.055 (3)	0.013 (3)	0.008 (3)	0.008 (3)
N3	0.056 (3)	0.047 (2)	0.054 (3)	−0.004 (2)	0.009 (2)	−0.004 (2)
C31	0.067 (4)	0.049 (3)	0.051 (3)	0.009 (3)	0.011 (3)	0.009 (2)
C32	0.056 (3)	0.063 (4)	0.054 (3)	−0.006 (3)	0.009 (3)	0.015 (3)
C33	0.040 (3)	0.064 (4)	0.070 (4)	−0.005 (2)	0.009 (3)	0.013 (3)
C34	0.059 (4)	0.057 (3)	0.062 (4)	0.004 (3)	0.011 (3)	0.010 (3)
N4	0.049 (3)	0.045 (2)	0.057 (3)	−0.010 (2)	0.006 (2)	0.006 (2)
C41	0.066 (4)	0.063 (4)	0.049 (3)	0.010 (3)	0.009 (3)	0.015 (3)
C42	0.047 (3)	0.057 (3)	0.054 (3)	−0.006 (2)	0.011 (2)	0.011 (3)
C43	0.050 (3)	0.056 (3)	0.050 (3)	−0.015 (2)	0.015 (2)	−0.011 (2)
C44	0.053 (3)	0.051 (3)	0.059 (3)	0.012 (3)	−0.005 (3)	0.009 (3)

### Geometric parameters (Å, °)

**Table:**

Mo1—S8	2.1589 (16)	C23—H23C	0.9600
Mo1—S7	2.1710 (17)	C24—H24A	0.9600
Mo1—S1	2.2013 (16)	C24—H24B	0.9600
Mo1—S2	2.2057 (15)	C24—H24C	0.9600
Mo1—Cu1	2.7241 (9)	N3—C33	1.401 (8)
Mo2—S6	2.1451 (17)	N3—C31	1.453 (8)
Mo2—S5	2.1742 (16)	N3—C32	1.549 (9)
Mo2—S4	2.2006 (17)	N3—C34	1.550 (6)
Mo2—S3	2.2148 (16)	C31—H31A	0.9600
Mo2—Cu1	2.7241 (10)	C31—H31B	0.9600
Cu1—S3	2.2549 (17)	C31—H31C	0.9600
Cu1—S1	2.3161 (15)	C32—H32A	0.9600
Cu1—S2	2.3518 (16)	C32—H32B	0.9600
Cu1—S4	2.3630 (17)	C32—H32C	0.9600
O1—C11	1.232 (8)	C33—H33A	0.9600
N1—C11	1.338 (8)	C33—H33B	0.9600
N1—C12	1.434 (9)	C33—H33C	0.9600
N1—C13	1.447 (8)	C34—H34A	0.9600
C11—H11A	0.9300	C34—H34B	0.9600
C12—H12A	0.9600	C34—H34C	0.9600
C12—H12B	0.9600	N4—C41	1.392 (9)
C12—H12C	0.9600	N4—C42	1.481 (7)
C13—H13A	0.9600	N4—C44	1.481 (7)
C13—H13B	0.9600	N4—C43	1.515 (7)
C13—H13C	0.9600	C41—H41A	0.9600
N2—C24	1.443 (8)	C41—H41B	0.9600
N2—C22	1.473 (8)	C41—H41C	0.9600
N2—C21	1.478 (9)	C42—H42A	0.9600
N2—C23	1.508 (7)	C42—H42B	0.9600
C21—H21A	0.9600	C42—H42C	0.9600
C21—H21B	0.9600	C43—H43A	0.9600
C21—H21C	0.9600	C43—H43B	0.9600
C22—H22A	0.9600	C43—H43C	0.9600
C22—H22B	0.9600	C44—H44A	0.9600
C22—H22C	0.9600	C44—H44B	0.9600
C23—H23A	0.9600	C44—H44C	0.9600
C23—H23B	0.9600		
			
S8—Mo1—S7	111.79 (6)	H22B—C22—H22C	109.5
S8—Mo1—S1	107.55 (6)	N2—C23—H23A	109.5
S7—Mo1—S1	111.04 (6)	N2—C23—H23B	109.5
S8—Mo1—S2	110.59 (6)	H23A—C23—H23B	109.5
S7—Mo1—S2	108.54 (6)	N2—C23—H23C	109.5
S1—Mo1—S2	107.24 (6)	H23A—C23—H23C	109.5
S8—Mo1—Cu1	139.84 (5)	H23B—C23—H23C	109.5
S7—Mo1—Cu1	108.35 (5)	N2—C24—H24A	109.5
S1—Mo1—Cu1	54.87 (4)	N2—C24—H24B	109.5
S2—Mo1—Cu1	55.80 (4)	H24A—C24—H24B	109.5
S6—Mo2—S5	111.20 (6)	N2—C24—H24C	109.5
S6—Mo2—S4	109.02 (6)	H24A—C24—H24C	109.5
S5—Mo2—S4	108.98 (6)	H24B—C24—H24C	109.5
S6—Mo2—S3	108.88 (6)	C33—N3—C31	109.6 (5)
S5—Mo2—S3	109.52 (6)	C33—N3—C32	112.8 (5)
S4—Mo2—S3	109.21 (6)	C31—N3—C32	109.4 (5)
S6—Mo2—Cu1	126.23 (5)	C33—N3—C34	108.8 (4)
S5—Mo2—Cu1	122.54 (5)	C31—N3—C34	108.6 (4)
S4—Mo2—Cu1	56.14 (4)	C32—N3—C34	107.5 (4)
S3—Mo2—Cu1	53.12 (5)	N3—C31—H31A	109.5
S3—Cu1—S1	117.80 (6)	N3—C31—H31B	109.5
S3—Cu1—S2	115.59 (6)	H31A—C31—H31B	109.5
S1—Cu1—S2	98.94 (6)	N3—C31—H31C	109.5
S3—Cu1—S4	102.39 (6)	H31A—C31—H31C	109.5
S1—Cu1—S4	110.01 (6)	H31B—C31—H31C	109.5
S2—Cu1—S4	112.49 (6)	N3—C32—H32A	109.5
S3—Cu1—Mo2	51.78 (4)	N3—C32—H32B	109.5
S1—Cu1—Mo2	132.00 (5)	H32A—C32—H32B	109.5
S2—Cu1—Mo2	128.60 (4)	N3—C32—H32C	109.5
S4—Cu1—Mo2	50.66 (4)	H32A—C32—H32C	109.5
S3—Cu1—Mo1	147.85 (5)	H32B—C32—H32C	109.5
S1—Cu1—Mo1	51.01 (4)	N3—C33—H33A	109.5
S2—Cu1—Mo1	50.87 (4)	N3—C33—H33B	109.5
S4—Cu1—Mo1	109.76 (5)	H33A—C33—H33B	109.5
Mo2—Cu1—Mo1	160.24 (3)	N3—C33—H33C	109.5
Mo1—S1—Cu1	74.13 (5)	H33A—C33—H33C	109.5
Mo1—S2—Cu1	73.34 (5)	H33B—C33—H33C	109.5
Mo2—S3—Cu1	75.09 (5)	N3—C34—H34A	109.5
Mo2—S4—Cu1	73.20 (5)	N3—C34—H34B	109.5
C11—N1—C12	122.4 (5)	H34A—C34—H34B	109.5
C11—N1—C13	119.2 (5)	N3—C34—H34C	109.5
C12—N1—C13	116.6 (5)	H34A—C34—H34C	109.5
O1—C11—N1	120.3 (5)	H34B—C34—H34C	109.5
O1—C11—H11A	119.9	C41—N4—C42	107.0 (3)
N1—C11—H11A	119.9	C41—N4—C44	112.8 (3)
N1—C12—H12A	109.5	C42—N4—C44	110.6 (4)
N1—C12—H12B	109.5	C41—N4—C43	108.4 (3)
H12A—C12—H12B	109.5	C42—N4—C43	108.3 (4)
N1—C12—H12C	109.5	C44—N4—C43	109.5 (4)
H12A—C12—H12C	109.5	N4—C41—H41A	109.5
H12B—C12—H12C	109.5	N4—C41—H41B	109.5
N1—C13—H13A	109.5	H41A—C41—H41B	109.5
N1—C13—H13B	109.5	N4—C41—H41C	109.5
H13A—C13—H13B	109.5	H41A—C41—H41C	109.5
N1—C13—H13C	109.5	H41B—C41—H41C	109.5
H13A—C13—H13C	109.5	N4—C42—H42A	109.5
H13B—C13—H13C	109.5	N4—C42—H42B	109.5
C24—N2—C22	108.6 (5)	H42A—C42—H42B	109.5
C24—N2—C21	115.1 (5)	N4—C42—H42C	109.5
C22—N2—C21	102.0 (4)	H42A—C42—H42C	109.5
C24—N2—C23	109.7 (5)	H42B—C42—H42C	109.5
C22—N2—C23	106.8 (5)	N4—C43—H43A	109.5
C21—N2—C23	113.9 (5)	N4—C43—H43B	109.5
N2—C21—H21A	109.5	H43A—C43—H43B	109.5
N2—C21—H21B	109.5	N4—C43—H43C	109.5
H21A—C21—H21B	109.5	H43A—C43—H43C	109.5
N2—C21—H21C	109.5	H43B—C43—H43C	109.5
H21A—C21—H21C	109.5	N4—C44—H44A	109.5
H21B—C21—H21C	109.5	N4—C44—H44B	109.5
N2—C22—H22A	109.5	H44A—C44—H44B	109.5
N2—C22—H22B	109.5	N4—C44—H44C	109.5
H22A—C22—H22B	109.5	H44A—C44—H44C	109.5
N2—C22—H22C	109.5	H44B—C44—H44C	109.5
H22A—C22—H22C	109.5		
			
S6—Mo2—Cu1—S3	87.24 (8)	S7—Mo1—Cu1—Mo2	3.80 (11)
S5—Mo2—Cu1—S3	−90.96 (7)	S1—Mo1—Cu1—Mo2	107.05 (11)
S4—Mo2—Cu1—S3	176.95 (6)	S2—Mo1—Cu1—Mo2	−96.54 (10)
S6—Mo2—Cu1—S1	−7.91 (9)	S8—Mo1—S1—Cu1	−139.25 (6)
S5—Mo2—Cu1—S1	173.89 (8)	S7—Mo1—S1—Cu1	98.15 (6)
S4—Mo2—Cu1—S1	81.80 (8)	S2—Mo1—S1—Cu1	−20.28 (6)
S3—Mo2—Cu1—S1	−95.15 (8)	S3—Cu1—S1—Mo1	143.57 (6)
S6—Mo2—Cu1—S2	−178.47 (8)	S2—Cu1—S1—Mo1	18.32 (6)
S5—Mo2—Cu1—S2	3.34 (9)	S4—Cu1—S1—Mo1	−99.67 (6)
S4—Mo2—Cu1—S2	−88.76 (8)	Mo2—Cu1—S1—Mo1	−154.22 (5)
S3—Mo2—Cu1—S2	94.29 (8)	S8—Mo1—S2—Cu1	137.04 (6)
S6—Mo2—Cu1—S4	−89.71 (8)	S7—Mo1—S2—Cu1	−99.99 (6)
S5—Mo2—Cu1—S4	92.10 (7)	S1—Mo1—S2—Cu1	20.04 (6)
S3—Mo2—Cu1—S4	−176.95 (6)	S3—Cu1—S2—Mo1	−145.14 (5)
S6—Mo2—Cu1—Mo1	−98.05 (11)	S1—Cu1—S2—Mo1	−18.36 (6)
S5—Mo2—Cu1—Mo1	83.75 (11)	S4—Cu1—S2—Mo1	97.75 (6)
S4—Mo2—Cu1—Mo1	−8.35 (10)	Mo2—Cu1—S2—Mo1	154.55 (4)
S3—Mo2—Cu1—Mo1	174.71 (11)	S6—Mo2—S3—Cu1	−121.63 (6)
S8—Mo1—Cu1—S3	−5.97 (12)	S5—Mo2—S3—Cu1	116.59 (6)
S7—Mo1—Cu1—S3	175.98 (9)	S4—Mo2—S3—Cu1	−2.69 (6)
S1—Mo1—Cu1—S3	−80.77 (10)	S1—Cu1—S3—Mo2	123.21 (6)
S2—Mo1—Cu1—S3	75.63 (10)	S2—Cu1—S3—Mo2	−120.22 (5)
S8—Mo1—Cu1—S1	74.81 (8)	S4—Cu1—S3—Mo2	2.42 (5)
S7—Mo1—Cu1—S1	−103.25 (7)	Mo1—Cu1—S3—Mo2	−176.64 (7)
S2—Mo1—Cu1—S1	156.40 (7)	S6—Mo2—S4—Cu1	121.44 (5)
S8—Mo1—Cu1—S2	−81.60 (8)	S5—Mo2—S4—Cu1	−117.02 (5)
S7—Mo1—Cu1—S2	100.35 (7)	S3—Mo2—S4—Cu1	2.59 (5)
S1—Mo1—Cu1—S2	−156.40 (7)	S3—Cu1—S4—Mo2	−2.46 (5)
S8—Mo1—Cu1—S4	175.01 (7)	S1—Cu1—S4—Mo2	−128.49 (5)
S7—Mo1—Cu1—S4	−3.04 (6)	S2—Cu1—S4—Mo2	122.26 (6)
S1—Mo1—Cu1—S4	100.20 (7)	Mo1—Cu1—S4—Mo2	177.01 (3)
S2—Mo1—Cu1—S4	−103.39 (6)	C12—N1—C11—O1	177.0 (6)
S8—Mo1—Cu1—Mo2	−178.14 (9)	C13—N1—C11—O1	13.0 (9)
